# TiO_2_ micro-flowers composed of nanotubes and their application to dye-sensitized solar cells

**DOI:** 10.1186/1556-276X-9-93

**Published:** 2014-02-24

**Authors:** Woong-Rae Kim, Hun Park, Won-Youl Choi

**Affiliations:** 1 Department of Metal and Materials Engineering, Gangneung-Wonju National University, Gangneung 210-720, South Korea; 2 Photovoltaic Research Department, Green Energy Research Institute, Hyundai Heavy Industries Co., Ltd, Yongin 446-912, South Korea; 3 Research Institute for Dental Engineering, Gangneung-Wonju National University, Gangneung 210-720, South Korea

**Keywords:** Dye-sensitized solar cells, TiO_2_ nanotube, Micro-flowers, Anodizing

## Abstract

TiO_2_ micro-flowers were made to bloom on Ti foil by the anodic oxidation of Ti-protruding dots with a cylindrical shape. Arrays of the Ti-protruding dots were prepared by photolithography, which consisted of coating the photoresists, attaching a patterned mask, illuminating with UV light, etching the Ti surface by reactive ion etching (RIE), and stripping the photoresist on the Ti foil. The procedure for the blooming of the TiO_2_ micro-flowers was analyzed by field emission scanning electron microscopy (FESEM) as the anodizing time was increased. Photoelectrodes of dye-sensitized solar cells (DSCs) were fabricated using TiO_2_ micro-flowers. Bare TiO_2_ nanotube arrays were used for reference samples. The short-circuit current (*J*_sc_) and the power conversion efficiency of the DSCs based on the TiO_2_ micro-flowers were 4.340 mA/cm^2^ and 1.517%, respectively. These values of DSCs based on TiO_2_ micro-flowers were higher than those of bare samples. The TiO_2_ micro-flowers had a larger surface area for dye adsorption compared to bare TiO_2_ nanotube arrays, resulting in improved *J*_sc_ characteristics. The structure of the TiO_2_ micro-flowers allowed it to adsorb dyes very effectively, also demonstrating the potential to achieve higher power conversion efficiency levels for DSCs compared to a bare TiO_2_ nanotube array structure and the conventional TiO_2_ nanoparticle structure.

## Background

Dye-sensitized solar cells (DSCs) have received much attention since Grätzel and O’Regan achieved a remarkable level of efficiency through their use of mesoporous TiO_2_ films as a photoanode for DSCs in 1991 [1]. DSCs have several advantages compared to Si or copper indium gallium selenide (CIGS) solar cells as follows: (a) DSCs can be fabricated with non-vacuum processes, as opposed to Si or CIGS solar cells. The use of non-vacuum equipment offers the possibility to reduce costs. (b) Wet etching processes such as saw damage etching and texturing, which are widely used in Si solar cells, are not required during the fabrication of DSCs. The fabrication of DSCs is thus simplified without a wet etching process. (c) Colorful DSCs can be easily fabricated because dyes have various colors according to their light absorption characteristics. Although DSCs have these merits, the relatively low power conversion efficiency has become the main cause which limits the commercialization of DSCs.

Several attempts to enhance the performance levels of dyes [2-12], photoelectrodes [13-30], counter cathodes [31-36], and electrolytes [3,31,37-41] have been attempted in an effort to obtain improved efficiency in DSCs. Among these efforts, increasing the surface area of the photoelectrodes and reducing the degree of charge recombination between the photoelectrodes and electrolytes have been shown to be critical factors when seeking to improve the power conversion efficiency of DSCs. The TiO_2_ nanoparticle structure has shown the best performance in DSCs [3]. However, structural disorder, which exists at the contact point of TiO_2_ nanocrystalline particles, reportedly prohibits charge transport, resulting in limited photocurrents [27-29].

The effort to find alternative TiO_2_ nanostructures has been an important issue to researchers who attempt to increase the power conversion efficiency of DSCs. Various types of nanotechnologies have been applied to alternative TiO_2_ nanostructures such as nanorods [13], nanowires [14,15], nanotubes [16,18,19,22,23,25,27-30,42], [43], nanohemispheres [21,24], and nanoforests [17,20]. These structures were used to increase the surface area for dye adsorption and to facilitate charge transport through TiO_2_ films. Of these nanostructures, the TiO_2_ nanotube structure has the best potential to overcome the limitations of the TiO_2_ nanoparticle structure. A previous report showed that the electronic lifetimes of TiO_2_ nanotube-based DSCs were longer than those of TiO_2_ nanoparticle-based DSCs [30]. Due to the one-dimensional structure of TiO_2_ nanotube arrays, charge percolation in TiO_2_ nanotube-based films is easier than it is in the TiO_2_ nanoparticle structure [27-29].

In this study, TiO_2_ micro-flowers composed of nanotubes were fabricated by means of dot patterning, Ti etching, and anodizing methods. The dot patterning and etching of Ti substrates increased the anodizing area to form TiO_2_ nanotubes. By controlling the anodizing time, beautiful TiO_2_ micro-flowers were successfully made to bloom on Ti substrates and were applied to the photoelectrodes of DSCs. To the best of our knowledge, this is the first study to report the fabrication of TiO_2_ micro-flowers and their application to DSCs. The TiO_2_ micro-flower structure is strongly expected to enhance the possibility to overcome the limitations of the TiO_2_ nanoparticle structure.

## Methods

To fabricate the protruding dot patterns on a 0.5-mm-thick Ti foil (99%, Alfa Aesar Co., Ward Hill, MA, USA), 5-μm-thick negative photoresists (PR; L-300, Dongjin Co., Hwaseong-Si, South Korea) were coated on a flat layer of Ti foil using a spin coater (Mark-8 Track, TEL Co., Tokyo, Japan). The coated photoresists were softly baked at 120°C for 120 s and hardly baked at 110°C for 5 min. A dot-patterned photomask was used for PR, the patterning process via UV light exposure. UV light having an energy of 14.5 mJ/s was used for illumination for 5 s, and the PR were developed. The PR at areas not exposed to UV light were removed.

The PR-patterned Ti foil was dry-etched at 20°C for 30 min using reactive ion etching (RIE) equipment (ICP380, Oxford Co., Abingdon, Oxfordshire, UK). BCl_3_ and Cl_2_ were used as the etchant gas in the RIE process with a top power of 800 W and a bottom power of 150 W. The photoresists on the UV-exposed area served to protect the flat Ti surface during the RIE process. Only the Ti surface at the area not exposed to UV was etched out. The remaining photoresist after the RIE process was stripped at 250°C for 20 min using a photoresist stripper (TS-200, PSK Co., Hwaseong-si, South Korea). O_2_ and N_2_ gases were used to remove the photoresist at a power of 2,500 W.

Before the anodizing process, Ti foil samples patterned with protruding dots were successively sonicated with acetone, ethanol, and deionized (DI) water to remove any residue on their surfaces. TiO_2_ micro-flowers, consisting of TiO_2_ nanotubes, were fabricated by the anodization of the Ti foil sheets which had been patterned with protruding dots in an ethylene glycol solution containing 0.5 wt% NH_4_F. A constant potential of 60 V with a ramping speed of 1 V/s was applied between the anode and the cathode. Pt metal was used as a counter cathode. The anodizing time was controlled for the successful blooming of the TiO_2_ micro-flowers. The as-anodized TiO_2_ nanotubes were rinsed with DI water and annealed at 500°C for 1 h. The morphologies of the TiO_2_ nanotubes and the micro-flowers were studied by field emission scanning electron microscopy (FESEM, Hitachi SU-70, Tokyo, Japan). The as-anodized and annealed TiO_2_ nanotubes were analyzed by X-ray diffraction (XRD; Rigaku D/MAX-RC, Cu Kα radiation, Rigaku Corporation, Tokyo, Japan) to confirm the crystallization characteristics.

Ti substrates based on TiO_2_ micro-flowers were used for the photoelectrodes of the DSCs. TiO_2_ photoelectrodes were immersed at room temperature for approximately 1 day in an ethanol solution containing 3 × 10^-4^ M *cis*-bis(isothiocyanato)bis(2,2′-bipyridyl-4,4′-dicarboxylato)ruthenium(II) bis-tetrabutylammonium (N719) dye. The dye-adsorbed photoelectrodes were rinsed with an ethanol solution and dried at room temperature. Pt-coated fluorine-doped tin oxide (FTO) glass as a counter electrode was prepared by spin coating a 0.7 mM H_2_PtCl_6_ solution in 2-propanol at 500 rpm for 10 s followed by an annealing step at 380°C for 30 min. The dye-adsorbed photoelectrodes and the Pt-coated FTO glass samples were spaced using a 60-μm Surlyn® film (DuPont Co., Wilmington, DE, USA). The liquid electrolyte was prepared by dissolving 0.6 M 1-hexyl-2,3-dimethylimidazolium iodide (C6DMIm), 0.05 M iodine, 0.1 M lithium iodide, and 0.5 M 4-*tert*-butylpyridine in 3-methoxyacetonitrile. The *J*-*V* characteristics were measured under an AM 1.5 G condition (model 2400 source measure unit, Keithley Co., Cleveland, OH, USA). A 1,000-W Xenon lamp (91193, Oriel Co., Irvine, CA, USA) was used as a light source.

## Results and discussion

**Figure 1:**
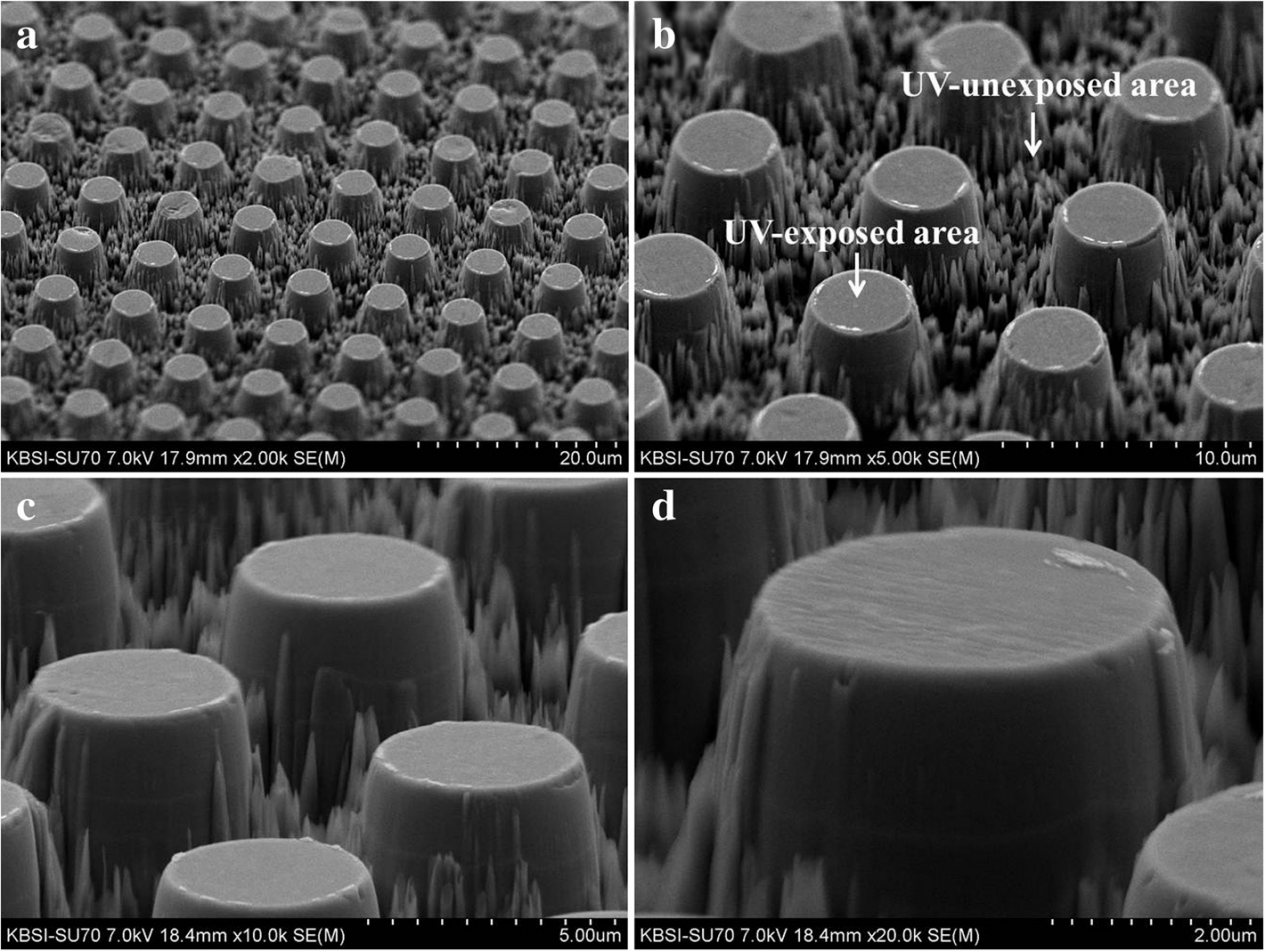
**FESEM images of a Ti surface patterned with protruding dots before the anodizing process. (a)** × 2,000 magnification, **(b)** × 5,000 magnification, **(c)** × 10,000 magnification, and **(d)** × 20,000 magnification.

Figure 1 shows FESEM images of Ti-protruding dots which have a cylindrical shape. The Ti surface at the UV-exposed area was flat because the cross-linked photoresist blocked the etching by reactive ions. However, the surface at the area not exposed to UV was very rough due to the RIE in the vertical direction. The diameter and height of the protruding dots were approximately 4 and 5 μm, respectively.

**Figure 2:**
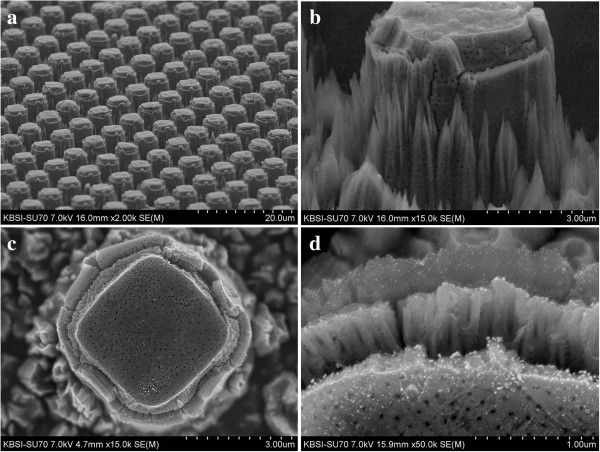
**FESEM images of a Ti surface patterned with protruding dots and anodized for 1 min.** The Ti surface was anodized at 60 V in an ethylene glycol solution containing 0.5 wt% NH_4_F. **(a)** × 2,000 magnification, **(b)** × 15,000 magnification, **(c)** × 15,000 magnification, and **(d)** × 50,000 magnification.

**Figure 3:**
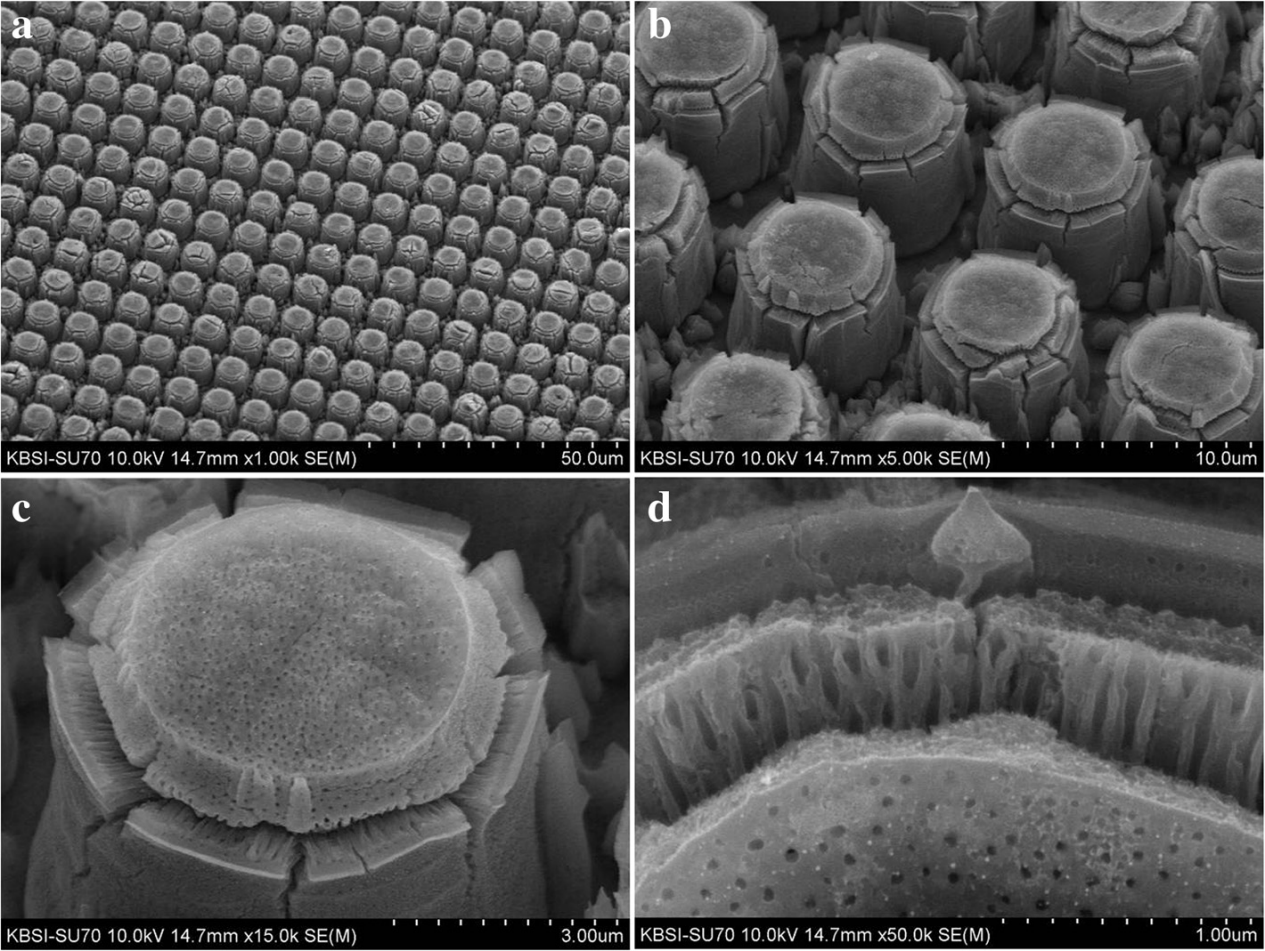
**FESEM images of a Ti surface patterned with protruding dots and anodized for 2 min.** The Ti surface was anodized at 60 V in an ethylene glycol solution containing 0.5 wt% NH_4_F. **(a)** × 1,000 magnification, **(b)** × 5,000 magnification, **(c)** × 15,000 magnification, and **(d)** × 50,000 magnification.

**Figure 4:**
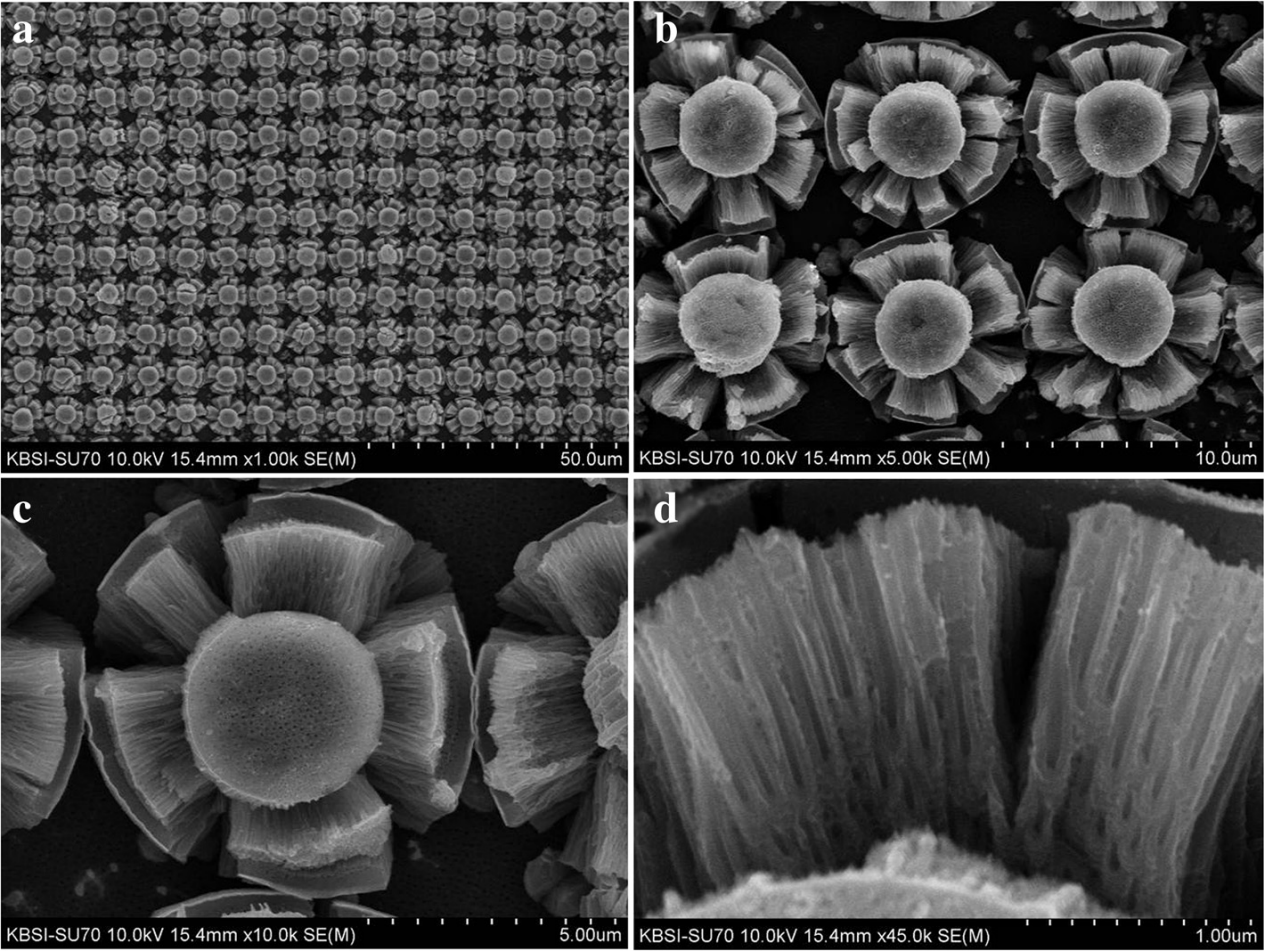
**FESEM images of a Ti surface patterned with protruding dots and anodized for 4 min.** The Ti surface was anodized at 60 V in an ethylene glycol solution containing 0.5 wt% NH_4_F. **(a)** × 1,000 magnification, **(b)** × 5,000 magnification, **(c)** × 10,000 magnification, and **(d)** × 45,000 magnification.

**Figure 5:**
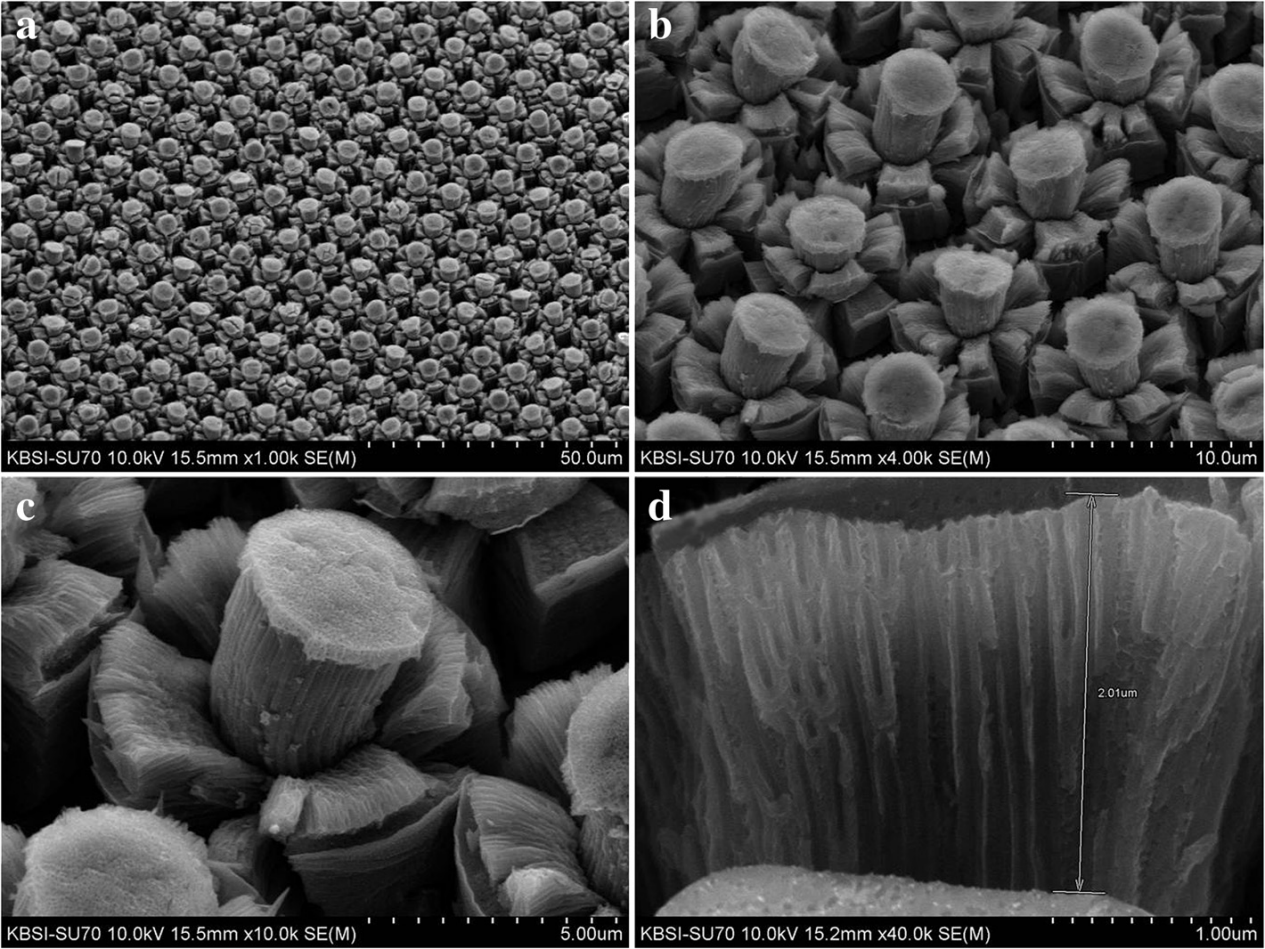
**FESEM images of a Ti surface patterned with protruding dots and anodized for 5 min.** The Ti surface was anodized at 60 V in an ethylene glycol solution containing 0.5 wt% NH_4_F. **(a)** × 1,000 magnification, **(b)** × 4,000 magnification, **(c)** × 10,000 magnification, and **(d)** × 40,000 magnification.

**Figure 6:**
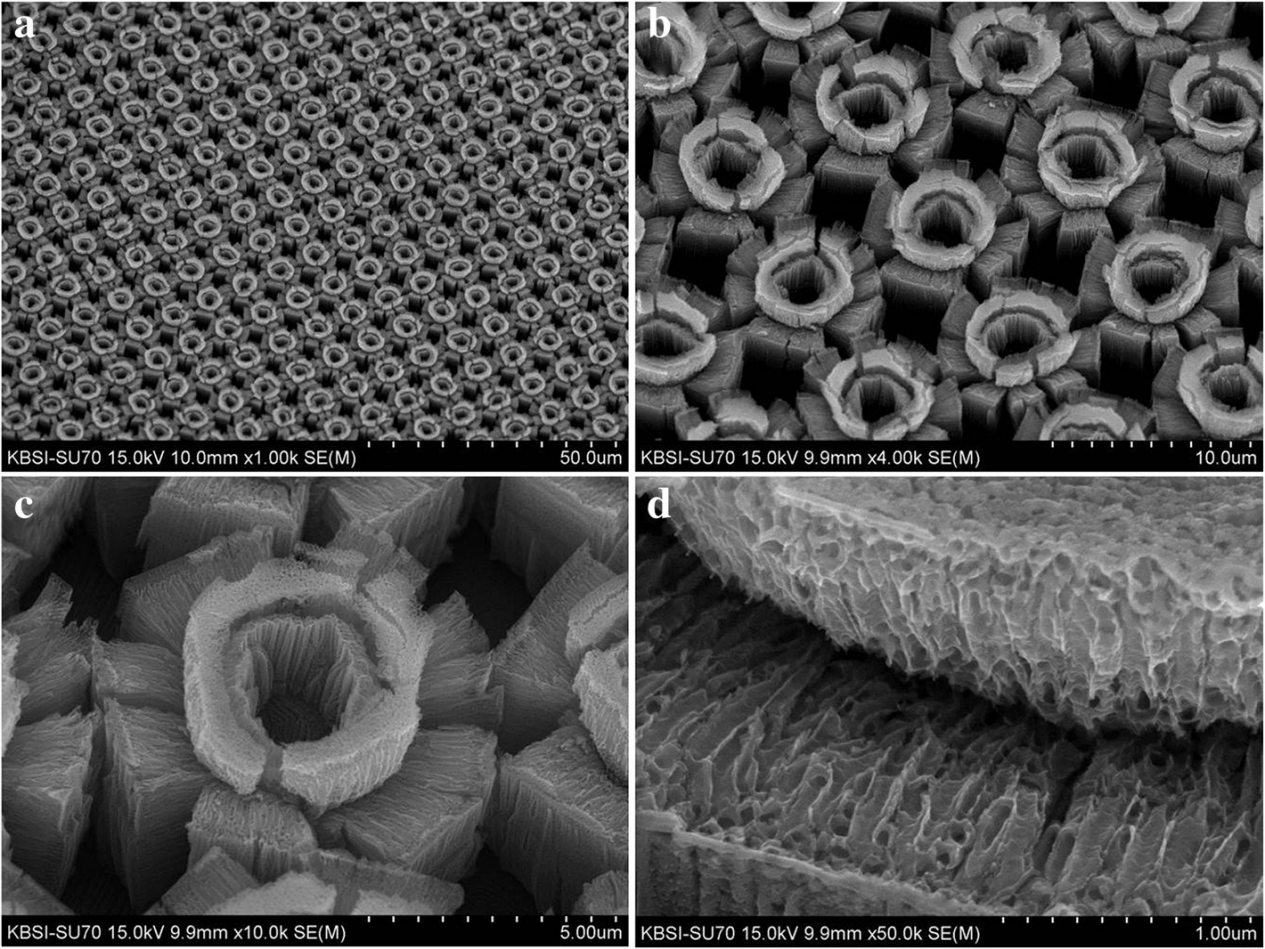
**FESEM images of a Ti surface patterned with protruding dots and anodized for 7 min.** The Ti surface was anodized at 60 V in an ethylene glycol solution containing 0.5 wt% NH_4_F. **(a)** × 1,000 magnification, **(b)** × 4,000 magnification, **(c)** × 10,000 magnification, and **(d)** × 50,000 magnification.

The microstructures while increasing the anodization time from 1 to 7 min are shown in Figures 2, 3, 4, 5, and 6. Figure 2 shows FESEM images of a Ti surface which was patterned with protruding dots and anodized for 1 min at 60 V in an ethylene glycol solution containing 0.5 wt% NH_4_F. Anodized Ti dot arrays are shown in Figure 2a, and magnified images of an anodized Ti dot are shown in Figure 2b,c. Several holes were formed on the top and the wall of the protruding dots. TiO_2_ nanotubes with a thickness of 400 nm were noted on the wall of the protruding dots, as shown in Figure 2d. Fluorine ions in the anodizing solution anisotropically etched the Ti and TiO_2_ due to the applied voltage between the anode and cathode. The anisotropic etching of Ti and TiO_2_ led to the creation of the one-dimensional structure of a TiO_2_ nanotube array. Figure 2d shows that the TiO_2_ nanotubes grew vertically from the wall of the protruding dots. When the anodization time was increased to 2 min, small cleavages formed between the top areas and side walls of the protruding dots, as shown in Figure 3. Figure 3b,c shows approximately 700-nm-thick TiO_2_ nanotube arrays.

When the anodization time was increased to 4 min, beautiful TiO_2_ micro-flowers started to bloom. The arrays of TiO_2_ micro-flowers are shown in Figure 4a. The thickness of each TiO_2_ nanotube is linearly correlated with the extent to which the TiO_2_ micro-flowers bloom. The blooming of the TiO_2_ micro-flowers is due to the severe cleavages of the TiO_2_ nanotubes between the top areas and the side walls of the protruding dots. As the anodization time was increased to 5 min, core bundles of nanotubes in TiO_2_ micro-flowers were slightly bent in random directions, as shown in Figure 5a,b,c,d. This occurred due to the difference in the growing speed of each TiO_2_ nanotube in the core bundles. The measured thickness of the TiO_2_ nanotubes in Figure 5d was 2 μm. As the anodization time was increased to 7 min, the center area of the core nanotube bundles in the TiO_2_ micro-flowers was removed, as shown in Figure 6a,b,c. Figure 6d shows the cleavage areas of the TiO_2_ micro-flowers. The structure of the TiO_2_ nanotubes in that area collapsed due to the additional etching by the fluorine ions in the anodizing solution.

**Figure 7:**
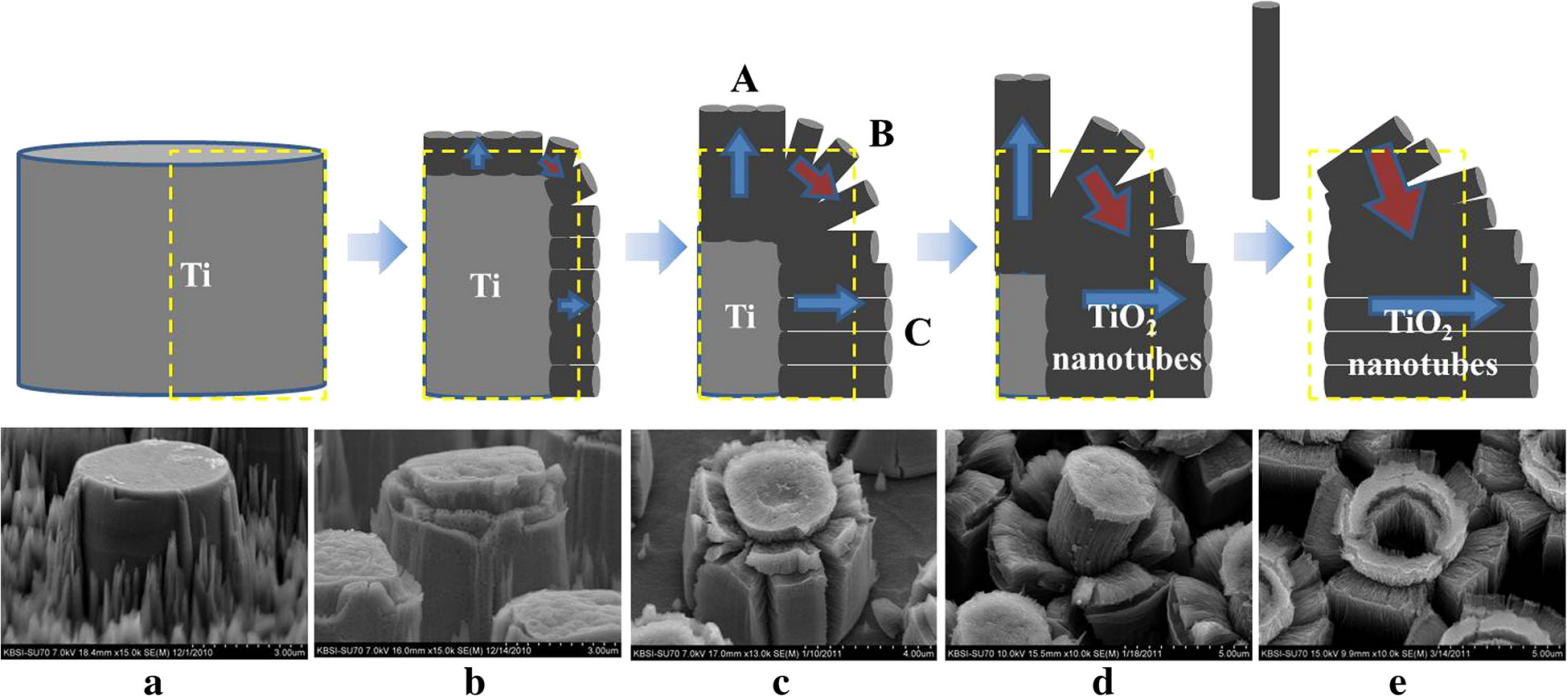
**Schematic mechanism for blooming of TiO**_**2 **_**micro-flowers with anodizing time. (a)** 0 min, **(b)** 1 min, **(c)** 3 min, **(d)** 5 min, and **(e)** 7 min.

Figure 7 shows the schematic mechanism involved in the blooming of the TiO_2_ micro-flowers. One of the Ti-protruding dots from the photolithography and RIE process shows a cylindrical shape in Figure 7a. Figure 7b shows that the TiO_2_ nanotubes grew in a vertical direction from the Ti surface due to the anodizing process. As the thicknesses of the TiO_2_ nanotubes at the cylindrical upper side (area A) and at the cylinder side (area C) increased, the Ti-supporting metal at the cylinder corner (area B) was completely converted into TiO_2_ nanotubes. The TiO_2_ nanotubes without Ti-supporting metal in area B finally fell onto the TiO_2_ nanotubes which had grown in area C, as shown in Figure 7c. Several horizontal cleavages in area B formed due to the collapse of the TiO_2_ nanotubes in area B. Several vertical cleavages in areas B and C were also observed, resulting from the volume expansion when the Ti was converted into TiO_2_ nanotubes. Volume expansion in an organic anodizing solution was reported previously [44]. Figure 7d shows that the growing TiO_2_ nanotubes in area C pushed and pushed TiO_2_ nanotubes between areas A and B to area C. More horizontal cleavages in area B were created due to the pushing of the TiO_2_ nanotubes, and these cleavages formed the multi-layered petals in the TiO_2_ micro-flowers. Figure 7c,d shows the blooming of beautiful TiO_2_ micro-flowers. This is a first blooming of TiO_2_ micro-flowers. The thickness of the TiO_2_ nanotubes in areas A and C gradually increased with the anodization time. Finally, all Ti metal was converted into TiO_2_ nanotubes, leaving no additional Ti metal to support the TiO_2_ nanotubes in area A. Figure 7e shows that the TiO_2_ nanotubes without Ti-supporting metal in area A were detached from the center of the nanotube bundles. This removal of the TiO_2_ nanotubes in area A left an empty core in the TiO_2_ micro-flowers. These TiO_2_ micro-flowers with empty cores are different from those shown in Figure 7c,d. This result represents a second blooming of the TiO_2_ micro-flowers.

**Figure 8:**
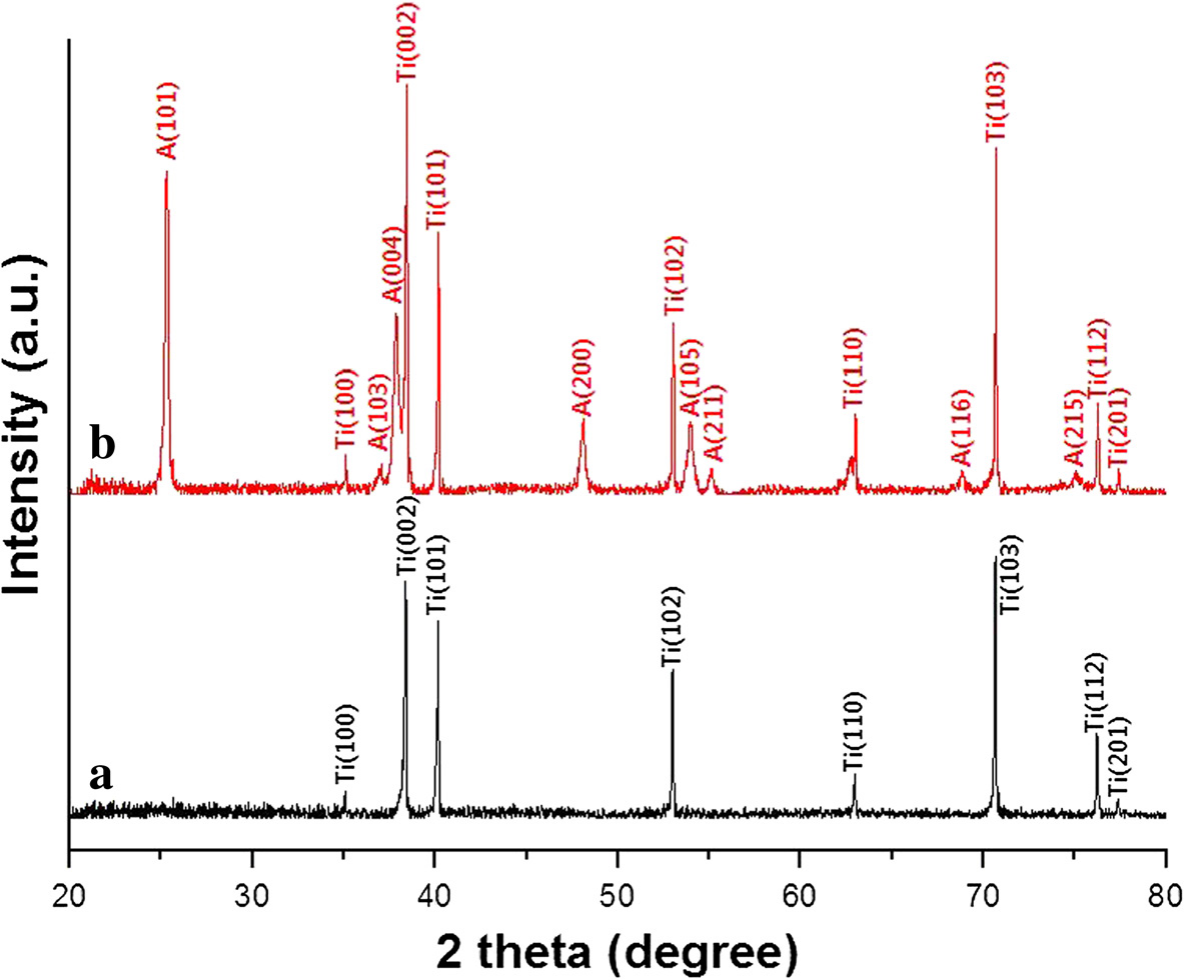
**XRD analysis of (a) as-anodized TiO**_**2 **_**micro-flowers and (b) annealed TiO**_**2 **_**micro-flowers.**

Figure 8 shows the results of an XRD analysis of the as-anodized TiO_2_ micro-flowers and the annealed TiO_2_ micro-flowers. Figure 8a shows only the Ti peaks, revealing that the as-anodized TiO_2_ nanotubes in the micro-flowers have an amorphous crystal structure. However, if the as-anodized TiO_2_ nanotubes are annealed at 500°C for 1 h, the crystal structure of the TiO_2_ nanotubes is converted into the anatase phase. Anatase peaks and Ti peaks were found, as shown in Figure 8b. From the XRD results, it can be confirmed that the annealed TiO_2_ micro-flowers exist in the anatase phase.

**Figure 9:**
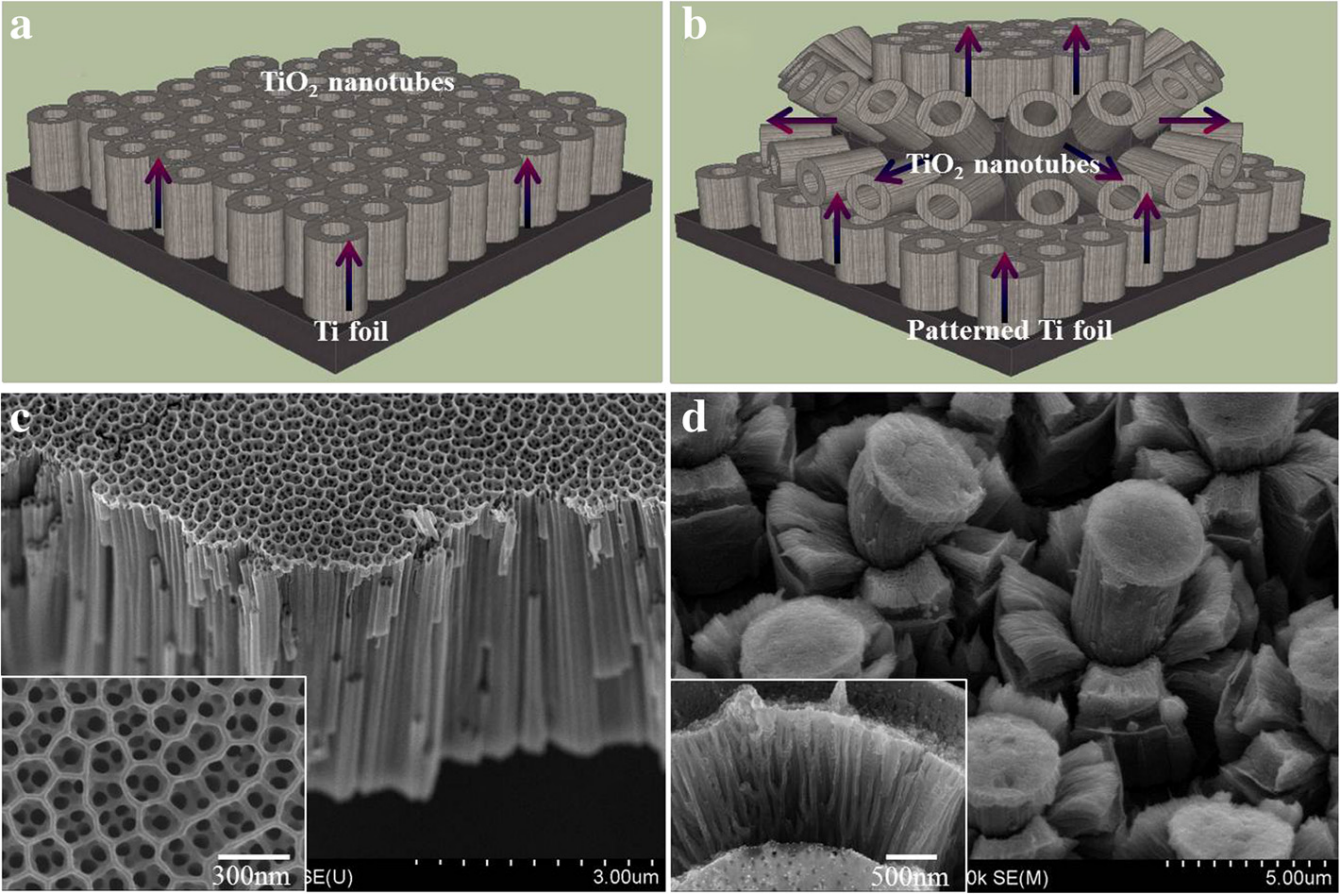
**Illustrations and FESEM images.** Illustrations of **(a)** bare TiO_2_ nanotube arrays and **(b)** TiO_2_ micro-flowers for a DSC photoelectrode. FESEM images of **(c)** bare TiO_2_ nanotube arrays and **(d)** TiO_2_ micro-flowers.

As shown in Figure 9, bare TiO_2_ nanotubes and TiO_2_ micro-flowers were applied for use in DSC photoelectrodes. DSCs based on bare TiO_2_ nanotube arrays were used as reference samples to compare the *J*-*V* characteristics with DSCs based on TiO_2_ micro-flowers. Photoelectrodes based on bare TiO_2_ nanotubes were prepared by an anodizing process of flat Ti foil. On the other hand, photoelectrodes based on TiO_2_ micro-flowers were fabricated by an anodizing process of Ti foil patterned and shaped such that they approximated cylindrical protruding dots.

**Figure 10:**
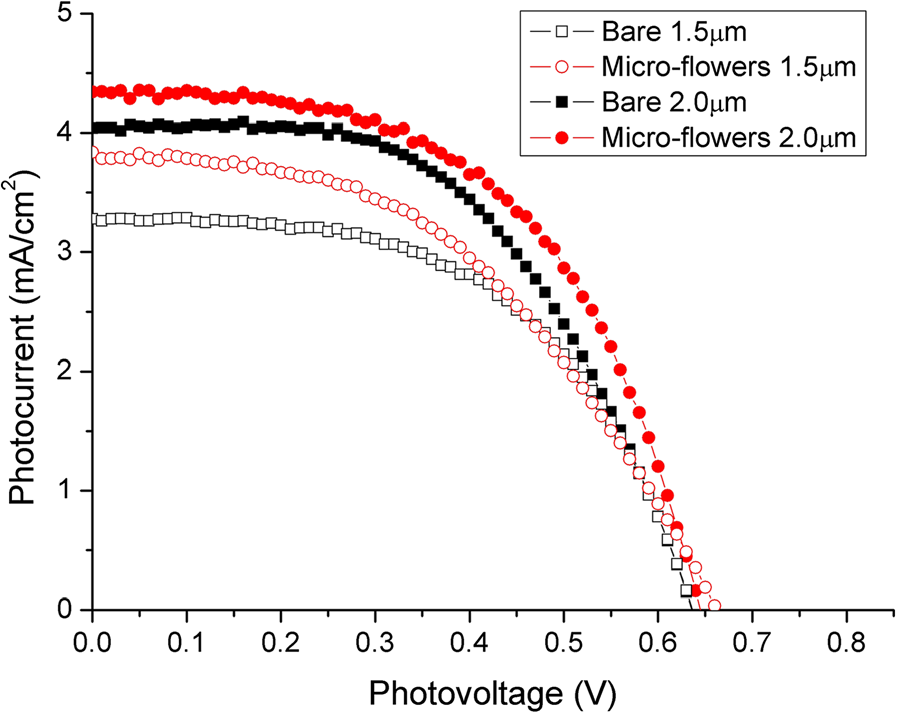
***J*-*V *characteristics of DSCs based on bare TiO**_**2 **_**nanotubes and TiO**_**2 **_**micro-flowers.** The thicknesses of the TiO_2_ nanotubes are 1.5 and 2.0 μm.

Figure 10 shows the *J*-*V* characteristics of DSCs based on the bare TiO_2_ nanotubes and TiO_2_ micro-flowers when the thicknesses of the TiO_2_ nanotubes are 1.5 and 2.0 μm, respectively. When the thickness of the TiO_2_ nanotubes was 1.5 μm, the short-circuit current (*J*_sc_), open-circuit voltage (*V*_oc_), and power conversion efficiency of the DSCs based on the TiO_2_ micro-flowers were slightly higher than those of the bare TiO_2_ nanotubes, as shown in Figure 10 and Table 1. However, the fill factor of the samples based on the TiO_2_ micro-flowers showed a decrease compared to that of the bare samples. When the thickness of the TiO_2_ nanotubes was increased from 1.5 to 2.0 μm, the *J*_sc_ of the DSCs based on the TiO_2_ micro-flowers increased from 3.838 to 4.340 mA/cm^2^. This appears that the improvement of *J*_sc_ in the TiO_2_ micro-flower samples is due to the increased surface area for dye adsorption. The efficiency of DSCs based on TiO_2_ micro-flowers reached 1.517%. The obtained efficiency levels were relatively low, as the thicknesses of the TiO_2_ nanotubes were very thin at 1.5 and 2.0 μm. The thickness of the TiO_2_ nanoparticle layer in the conventional DSCs was approximately 20 μm. If the thickness of the TiO_2_ micro-flowers is increased, its efficiency will also increase. The performance levels of DSCs based on these TiO_2_ micro-flowers will also improve if the morphologies of the protruding dots, such as the dot diameter, the distance between adjacent dots, and the height of the cylindrical protrusions, are tailored. Our future work will concentrate on all of these factors to attain the maximum efficiency level from DSCs based on TiO_2_ micro-flowers. The conclusion of this report is that DSCs based on TiO_2_ micro-flowers have the potential to achieve higher efficiency levels compared to DSCs based on normal TiO_2_ nanotubes and TiO_2_ nanoparticles.

**Table 1:** ***J*-*V *** **characteristics of DSCs based on bare TiO**_**2 **_**nanotubes and TiO**_**2 **_**micro-flowers**

**Sample**	**Photoelectrode**	**Thickness of the TiO**_**2 **_**nanotubes (μm)**	***J***_**sc**_	***V***_**oc**_	**FF**	**Efficiency (%)**
			**(mA/cm**^**2**^**)**	**(V)**		
(a)	Bare	1.5	3.279	0.636	0.549	1.147 ± 0.167
(b)	Micro-flowers	1.5	3.838	0.661	0.467	1.187 ± 0.041
(c)	Bare	2.0	4.030	0.636	0.536	1.378 ± 0.092
(d)	Micro-flowers	2.0	4.340	0.644	0.542	1.517 ± 0.063

The thicknesses of TiO_2_ nanotubes are 1.5 μm and 2.0 μm.

## Conclusion

Ti-protruding dots with a cylindrical shape were fabricated by coating with a photoresist, illuminating with UV light, etching the Ti surface via the RIE method, and stripping the photoresist on Ti foil. When the Ti-protruding dots were anodized for over 3 min, beautiful arrays of TiO_2_ micro-flowers successfully bloomed on the Ti foil sheets. The blooming TiO_2_ micro-flowers were applied as the photoelectrodes of DSCs. The *J*-*V* characteristics of the DSCs based on the TiO_2_ micro-flowers were compared with those based on bare TiO_2_ nanotubes. The *J*_sc_ and power conversion efficiency values of DSCs based on TiO_2_ micro-flowers were higher than those of bare samples. TiO_2_ micro-flowers facilitated better dye adsorption, resulting in higher *J*_sc_ values. The TiO_2_ micro-flowers had a larger surface area for dye adsorption compared to that of bare TiO_2_ nanotubes. The efficiency of the DSCs based on the TiO_2_ micro-flowers was found to reach 1.517%. The efficiency levels of the DSCs based on the TiO_2_ micro-flowers were relatively low compared to those of conventional DSCs based on TiO_2_ nanoparticle structures, as the thickness of the TiO_2_ nanotubes in the micro-flowers was very small. To improve the efficiency of DSCs based on TiO_2_ micro-flowers, our future work will concentrate on controlling the characteristics of the dot patterns such as the dot diameter, the distance between adjacent dots, and the height of the protruding dots.

## Competing interests

The authors declare that they have no competing interests.

## Authors’ contributions

WK and HP conceived the study and drafted the manuscript. WK helped with the anodization of Ti surface. HP helped with the *J*-*V* characterization of DSCs. WC supervised the whole work and revised the manuscript. All authors read and approved the final manuscript.
